# Hyperspectral Sensor Data Capability for Retrieving Complex Urban Land Cover in Comparison with Multispectral Data: Venice City Case Study (Italy)

**DOI:** 10.3390/s8053299

**Published:** 2008-05-20

**Authors:** Rosa Maria Cavalli, Lorenzo Fusilli, Simone Pascucci, Stefano Pignatti, Federico Santini

**Affiliations:** 1 National Research Council, Institute of Atmospheric Pollution, Via Fosso del Cavaliere, 100, Roma, 00133, Italy; 2 National Research Council, Institute of Methodologies for Environmental Analysis, C.da S. Loja -Zona Industriale, Tito Scalo (PZ), 85050, Italy

**Keywords:** urban environmental monitoring, satellite hyperspectral remote sensing, object-oriented classification, band-depth analysis, linear spectral unmixing

## Abstract

This study aims at comparing the capability of different sensors to detect land cover materials within an historical urban center. The main objective is to evaluate the added value of hyperspectral sensors in mapping a complex urban context. In this study we used: (a) the ALI and Hyperion satellite data, (b) the LANDSAT ETM+ satellite data, (c) MIVIS airborne data and (d) the high spatial resolution IKONOS imagery as reference. The Venice city center shows a complex urban land cover and therefore was chosen for testing the spectral and spatial characteristics of different sensors in mapping the urban tissue. For this purpose, an object-oriented approach and different common classification methods were used. Moreover, spectra of the main anthropogenic surfaces (i.e. roofing and paving materials) were collected during the field campaigns conducted on the study area. They were exploited for applying band-depth and sub-pixel analyses to subsets of Hyperion and MIVIS hyperspectral imagery. The results show that satellite data with a 30m spatial resolution (ALI, LANDSAT ETM+ and HYPERION) are able to identify only the main urban land cover materials.

## 1. Introduction

Urban areas are currently the most rapidly changing types of land covers, even though they represent only a low percentage of the global land surface [32]. Their monitoring is one of the most relevant issues concerning the evaluation of the human impact on the environment. For this purpose, remote sensing imagery can provide a timely and synoptic view of urban land covers, as well as a tool to monitor changes in urbanizing landscapes. The most common approach for characterizing urban environments using remote sensing imagery are the land-cover and land-use classifications [6,13,36]. However, the remote sensing characterization of urban environments can be complicated for several reasons: (*i*) urban land-cover classes are not well spectrally distinct, resulting in considerable confusion between classes [36,38-40], (*ii*) the physical structure of land-use classes varies from site to site due to the different roofing and paving materials and building typology [8,21,32,34], (*iii*) urban areas are heterogeneous and most pixels, at the satellite spatial resolution of 30 m/pixel, appear mixed with varying proportions of different components and/or materials [33].

The potentialities offered by the new generation of hyperspectral satellite imagery for urban applications is a challenging aspect that this paper intends to deal with, as it is still not fully investigated [30,40].

A detailed knowledge of the spectral characteristics of urban surfaces is required for a successful identification of the surface materials from processed hyperspectral imagery. To this aim, spectroscopic studies, laboratory and field investigations have been conducted by many authors [2,15-18]. Spectral library analyses and studies [2,19] revealed that the spectral diversification of the urban surface materials is an important prerequisite for their identification. Several studies have illustrated the basic potential of airborne hyperspectral data and the new challenges of such data for the spectral differentiation of urban surface materials [2,15,16,19]. For example, Bokoye and Dionn [5] used satellite Hyperion data on the Montreal downtown stressing a general potential of spaceborne hyperspectral for urban space characterization.

In order to highlight the present interest on satellite hyperspectral potential for urban applications, it is noteworthy to recall the initiatives carried on in the recent years by the National Space Agencies (e.g., NASA, ESA, ASI and DLR) for the deployment of an operative hyperspectral spaceborne mission [12,31]. Within this framework, this study takes effort from a proposal submitted to the EO-1 Science Team to evaluate the Hyperion land cover mapping performances in different disciplines including the urban mapping [31,45,11]. In such context, complementing scenes of LANDSAT-ETM+ (http://landsat.gsfc.nasa.gov/) [47] and IKONOS (http://geo.arc.nasa.gov/) satellite data and of the airborne Multispectral Infrared Visible Imaging Spectrometer (MIVIS) data as well [1,4] were collected on the Venice (Italy) historical center test site.

More specific objectives for this study include the comparison of the ground-truth IKONOS imagery with (*i*) the hyperspectral Hyperion data, (*ii*) the multispectral satellite data (ALI and LANDSAT-ETM+), and (*iii*) the high spatial resolution hyperspectral airborne data (MIVIS). To this aim, an object-oriented approach, a clustering segmentation procedure and a common supervised classification method were used to classify the pre-processed datasets. Furthermore, the main spectral absorption features of the urban land cover occurring in the study area were analyzed and their detection limits were assessed. Next, a Band-Depth analysis was performed on Hyperion and MIVIS hyperspectral data taking into account the materials' detection limit results. Finally, sensors' efficiency in detecting fractional urban land cover abundances at the sub-pixel level was assessed through the application of unmixing algorithms.

## 2. Study area

Venice, the worldwide known city and relevant artistic centre, lies inside a large lagoon in northeastern Italy. The functional area of Venice is densely populated, with residential and industrial districts, grassy parks, paved squares and, of course, canals. The historical town is composed of about a hundred small islands, where buildings arise one close to the other, separated only by narrow streets (called “calli”), while the connection between the islands is guaranteed by several bridges.

**Figure 1.**
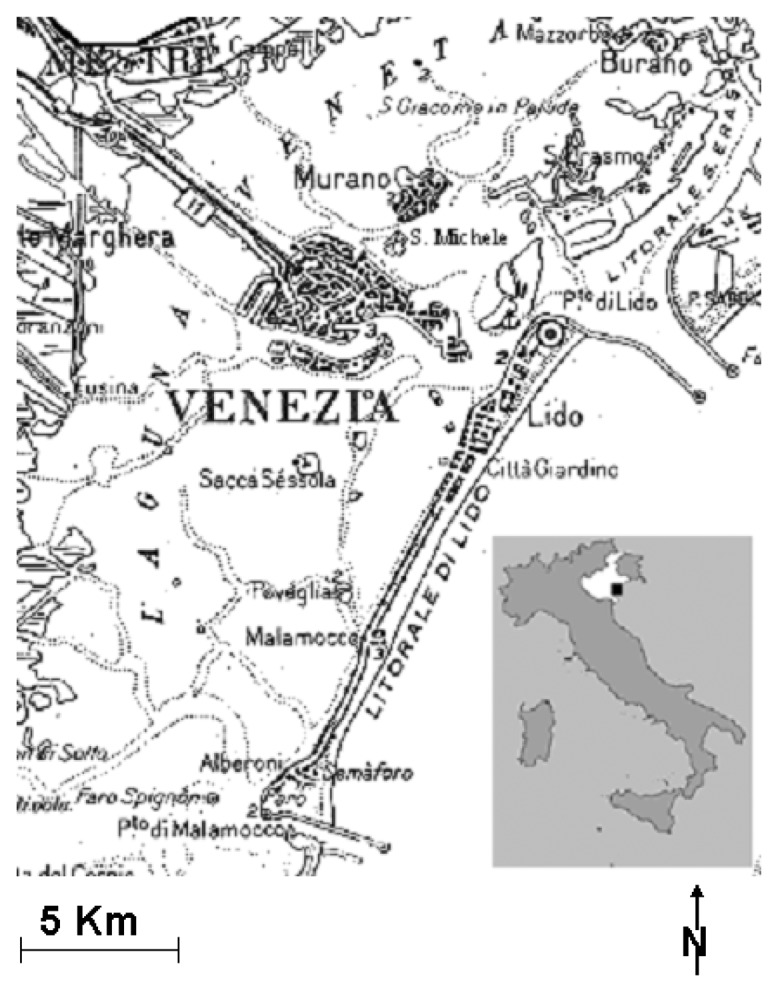
Location of the study area.

The Venice historical center was chosen as test site for its dense urban land covers that represent a suitable area on which verifying the potentiality of different sensors (i.e., different spectral and spatial resolution images) in mapping urban land covers at pixel and where feasible at sub-pixel scale. The study area (Figure 1) is characterized by a mixture of urban land cover types and surface materials.

## 3. Data

### 3.1. Remote sensing data

For this study, both satellite and airborne remote sensing data were processed. The main characteristics of the sensors are summarized in Table 1.

The Earth Observing-1 (EO-1) mission is carrying three advanced technology imaging instruments. They are the Advanced Land Imager (ALI), the Hyperion hyperspectral imager, and the LAC Atmospheric Corrector.

The ALI is designed to produce images directly comparable to those of the Enhanced Thematic Mapper Plus (ETM+) of Landsat 7. It employs novel wide-angle optics and a highly integrated multispectral and panchromatic spectrometer. Operating in a push-broom fashion at an orbit of 705 km, the ALI provides Landsat type panchromatic and multispectral bands. With a partially populated focal plane, the ALI wide-angle optics produces a ground swath image width of 37 km.

The focus of the Hyperion instrument is to provide high quality calibrated data that can support evaluation of hyperspectral technology for Earth observing missions. The Hyperion is a “push broom” instrument. Each image frame taken in this push-broom configuration captures the spectrum of a line 30 m long by 7.5 km wide (perpendicular to the satellite motion). It has a single telescope and two spectrometers, one VNIR spectrometer and one SWIR spectrometer. The telescope images the Earth onto a slit that defines the instantaneous field-of-view which is 0.624° wide (i.e., 7.5 km swath width from a 705 km altitude) by 42.55 m radians (30 meters) in the satellite velocity direction. Therefore, the Hyperion provides Earth imagery at 30 m spatial resolution and with a 7.5 km swath width in 220 contiguous spectral bands at 10 nm spectral resolution. Both ALI and Hyperion data were provided at Level 1R, i.e. radiometrically corrected with no geometric correction applied. The image data are provided in 16-bit radiance values.

The Landsat Enhanced Thematic Mapper Plus (ETM+) is a sensor carried onboard the Landsat 7 satellite and has acquired images of the Earth nearly continuously since July 1999, with a 16-day repeat cycle. Landsat ETM+ image data consist of eight spectral bands with a spatial resolution of 30 meters for bands 1 to 5 and band 7. Resolution for band 6 (thermal infrared) is 60 meters and resolution for band 8 (panchromatic) is 15 meters. The data were supplied at the Level 1R (L1R) data product that provides radiometrically corrected data where calibration is applied. Image data are not geometrically corrected or geographically referenced and are provided in 16-bit (radiance) values.

The IKONOS satellite acquires high-resolution push-broom imagery. IKONOS main characteristics are as follows: a sun synchronous orbit of 98.1 degree; an altitude of 681 Km; a resolution at Nadir of 0.82 m (panchromatic) and 3.2 m (multispectral); a ground resolution at 26° off-nadir of 1.0 m (panchromatic) and 4.0 m (multispectral); the image swath is 11.3 km at nadir and 13.8 Km at 26° off-nadir; the revisit time is approximately 3 days at 40° latitude and the dynamic range is 11-bits per pixel.

MIVIS hyperspectral sensor is a whisk-broom scanner with an axe head double mirror, it is a passive scanning and imaging instrument that is composed of 4 spectrometers which simultaneously record radiation coming from the Earth's surface. Summary specifications of MIVIS are as follows: 102 spectral bands from the VNIR to the TIR spectral range and wavelength range between 0.43-12.7 μm; an IFOV of 2 mrad and a digitized FOV of 71.1°; a numerisation (ADC) of 12 bits; scan rotational speed of 25, 16.7, 12.5, 8.3 and 6.25 scan/s; 2 reference black bodies selectable between 15°C and 45°C; a Position Attitude System composed of a GPS receiver for measuring aircraft's position (accuracy 15-20 m) and speed (accuracy 0.05-0.20 m/sec) and a gyroscope for determining aircraft's roll and pitch (accuracy ±0.2°) with a roll correction in real time between ±10°; a flux gate compass for finding aircraft's variation around the yaw axis (accuracy ±0.56°) and a computer-aided data quality check for all channels in real time.

The datasets acquired over Venice city consisted of (a) MIVIS airborne data acquired on July 26, 2001 at 10:45 (GMT), using scan rates of 25 scans/s at an altitude of 4000m, corresponding to a 8-m ground-pixel resolution at the instrument's IFOV; (b) EO-1 sensors satellite data acquired on June 7, 2001 at 11:56 (GMT); (c) Landsat ETM+ satellite data acquired on July 2, 2001; and (d) IKONOS satellite data acquired on July 2001.

As ALI sensor does not cover the entire Venice city (i.e. the western part of the city was out of the swath) all sensors comparisons were referred to the ALI scene.

For this study, the high spatial resolution IKONOS imagery (1m/pixel) of the Venice city was used as ground truth.

### 3.2. Image pre-processing

The satellite datasets with a 30m/pixel spatial resolution (i.e., ALI, ETM+ and Hyperion) were provided radiometrically calibrated at the sensor (Level 1R). Hyperion data were also corrected by the “SMILE” distortion effect by applying the method described in Datt et al. [11], and then processed using a global destriping procedure to reduce the spectral effects of column-to-column noise resulting from the pushbroom design [11]. The data volume was reduced to 157 bands, encompassing the 0.427 to 2.365 mm spectral region, by excluding noisy bands and the channels of water absorption.

The satellite data were corrected by atmospheric effects by means of the FLAASH module [29], as implemented in the ENVI 4.4. [26] software package, which incorporates the MODTRAN4 radiation transfer code [3]. The standard MODTRAN urban aerosol/haze type was selected [3] for the aerosol model as the visibility for all the images was high (i.e. greater than 40 km).

As regard MIVIS airborne data, the raw data were radiometrically calibrated to radiance (nW cm^-2^ sr^−1^ nm^−1^) using the calibration factors measured for each MIVIS channel, on the test bench, on June 2001 by the Italian CNR-LARA researchers [4]. Atmospheric correction procedures were applied to MIVIS radiance data using the MODTRAN radiative transfer code [3]. The MODTRAN code was used to calculate look-up tables of standard radiative functions to compute atmospheric correction (path radiance, atmospheric transmittance and solar flux) with respect to the variations in viewing and illumination angles, to the relative azimuth angle between scan lines, to the solar azimuth and terrain elevation. In the process, adjacency effects were considered using the empirical formula described in Vermote et al. [47]. The calibration results were validated using in situ spectral measurements (vegetation, bare soil, asphalt and different roof types) collected using the ASD field spectrometer.

In order to obtain a comparable reflectance data set, the residual errors occurring in the satellite data were minimized by applying the empirical line method, as implemented in the ENVI 4.4. [26] software package, by using the collected ground truth spectra. The empirical line calibration forces the image spectra to match reflectance spectra collected from the field. This method is capable of producing the most accurate results possible, but requires ground truth information.

The obtained reflectance values for Hyperion, ALI and LANDSAT ETM+ data sets were further geocoded in the UTM (Universal Transverse Mercator, European 1950 datum) map projection reference system, by using 30 Ground Control Points (GCPs) extracted from the Regional Technical Map at a scale of 1:10,000. An RMS error of about 0.9 pixels, accomplished in one step by means of the nearest-neighbor re-sampling algorithm, was obtained for the three sensors.

The IKONOS imagery, which was used as ground truth in this study, was geometrically corrected to the same UTM map projection as the other sensors. Moreover, in order to acquire a higher accuracy of the geocoded image, the rigorous model proposed by Toutin [44] was applied for the geometric correction method. A root mean square error approximately of 1-2 pixels (1.5m) was attained.

The geocoding process applied to MIVIS data was different, as the airborne images of a whiskbroom sensor are affected by geometric distortions. MIVIS data were geometrically corrected by using an own code, implemented in the IDL 4.4. software package [26], which is based on the precise trajectory reconstruction process by using onboard GPS/INS systems and additional ground control point information. In particular, MIVIS data were geocoded using (*i*) the sensor trajectory (sampled at 1Hz) and the platform attitude (sampled at 25Hz) recorded on board; (*ii*) the system whiskbroom geometry; (*iii*) a set of GCPs, extracted from the Regional Technical Map at a scale of 1:10,000, in the navigational data processing to reduce the uncertainties in the trajectory reconstruction. MIVIS images yielded an RMS error of 0.53 pixels.

At last, it must be considered that the comparison between the classified imagery and the IKONOS ground-truth reference can be influenced by the accuracy of the pixels location (RMS error) in those areas where a mixture of urban land cover types/surface materials occurs. The IKONOS ground truth cover vectorial map was further spatially resampled according to the sensor's spatial resolution to compare the retrieved covering materials abundance values.

### 3.3. Field campaign

Extensive field campaigns were conducted, from June to September 2001, using the portable field spectrometer FieldSpec FR Pro (ASDI Inc., Boulder, Colorado, USA). The ASD spectrometer samples a spectral range of 350–2500 nm using one detector spanning the VNIR and two spanning the SWIR, with a spectral sampling interval of 1.4 nm and 2.0 nm respectively for the VNIR and the SWIR.

The spectral analyses in the field were conducted (*i*) to distinguish the urban materials spectrum shape from other materials and backgrounds, (*ii*) to construct a spectral library of urban materials useful for calibrating and validating the remote sensing data and (*iii*) to provide urban material samples for laboratory analysis.

The field ASD measurements were collected within 2 hours of solar noon sets, acquiring 4-5 measures for each target from a height of 1 m using a field of view of 25° to fulfill the target dimension. Measurements were converted to absolute reflectance using NIST, calibrated panel (Spectralon reference standard).

**Figure 2.**
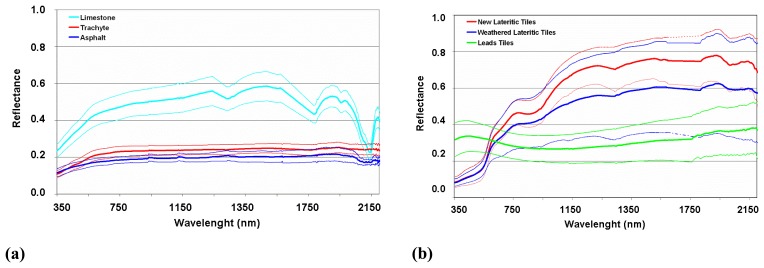
(a) ASD field spectra of limestone, asphalt and trachyte paving materials. (b) Spectra of new and weathered lateritic tiles and leads tiles (roofing materials). All spectra are plotted with the relative σ standard deviation.

On the basis of the field campaigns, six main materials were identified in the Venice historical center (Figure 2). They were: (a) the limestone (coming from the quarries of Pietra d'Istria, Italy) used for decoration in the urban paving; (b) the asphalt primarily used in the western site of the city and in the harbor areas; (c) the trachyte rock (coming from the quarry of the Colli Euganei, Italy) used for paving the pedestrian streets; (d,e) both new (light red color) and old (i.e., weathered and sometimes moss and lichen covered with dark red color) lateritic tiles; (f) lead tiles used as covering material for the public buildings and domes. Each urban building material was measured in different sites in order to sample the natural spectral variability (deviation standard of the collected measures) so determining a reflectance range of variability. Spectral measurements of the samples were also repeated in our laboratory for a better assessment of the spectral features of the material of interest.

The six main spectra of the material samples collected during the field campaigns are shown in Figure 2.

## 4. Methods

**Figure 3.**
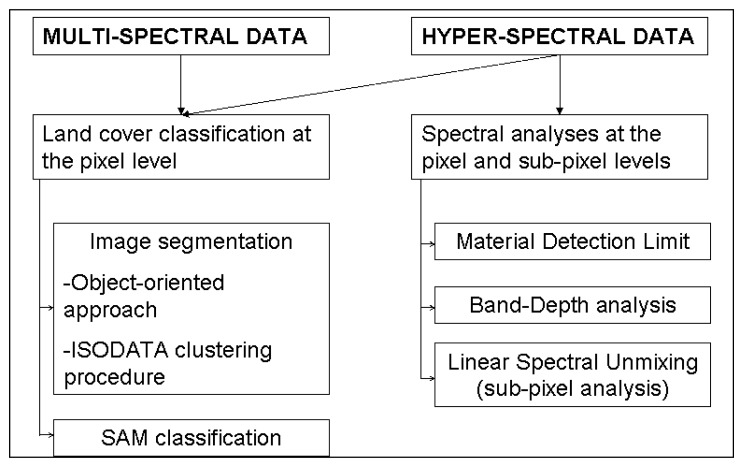
Flow diagram indicating the steps followed in the methods.

To verify the potentialities of the high spatial resolution and multi/hyperspectral sensors in distinguishing different surfacing materials in a complex urban environment, the following processing methodology was applied to the whole multi-sensor dataset. The procedure, illustrated in Figure 3, was implemented in the five steps detailed in the following section.

The information content of the Venice urban context was retrieved at the pixel level by applying: (a) an object-oriented approach and the ISODATA clustering procedure for the imagery segmentation and (b) the Spectral Angle Mapper (SAM) supervised classification method for classifying the land cover.

The high spectral resolution datasets (i.e., MIVIS and Hyperion) were further investigated to extract the fractional land cover information at the sub-pixel level. A preliminary (c) Band and Material Detection Limit (BDL, MDL) analyses [22,23] of the occurring urban materials were assessed in order to apply (d) a Band-Depth analysis. Finally, on the same areas, (e) a Linear Spectral Unmixing procedure was applied to both MIVIS and Hyperion data.

The thematic outputs from the different classification procedures were compared with respect to the vector ground truth derived by visual interpreting the multispectral IKONOS pansharpened imagery (1 m/pixel) obtained by applying the Intensity-Hue-Saturation (IHS) sharpening method, as implemented in the ENVI 4.4. [26] software package.

IKONOS imagery interpretation allowed to identify on the study area the following urban covering material percentages: limestone (0.64%), trachyte (13.08%), asphalt (6.71%), lateritic tiles (indistinctly new and weathered) (45.30%), lead tiles roofs (0.39%), vegetation (14.77%) and other minor materials (19.2%).

### 4.1. Image segmentation

The image segmentation procedure was performed in order to verify if its application can improve the results obtained by conventional pixel-based techniques, which consider only the specific spectral features of a given pixel without taking into account the spatial context of an object or an area [20,27,28,42].

A two-fold approach for the image segmentation was followed: *(i)* an object-oriented approach, and *(ii)* a clustering segmentation procedure, i.e. the standard ISODATA unsupervised classification method, which is based on the spectral information inherent to the sensor data.

#### 4.1.1. Object-Oriented approach

Since the spatial information is very important in classification processes to produce reliable maps [20,28,42], for this study we used an object-oriented approach with a segmentation procedure followed by classification as implemented in the Feature Extraction module of the ENVI 4.4 software package [26]. In more detail, the procedure consists of a combined process of segmenting the image into regions of pixels, computing attributes for each region to create objects, and last classifying the objects. To identify only urban land covers within the chosen study area, a workflow consisting of two main tasks was adopted. (*i*) The “*find objects*” task (i.e., segmentation, [20]) that was divided, in its turn, into four steps: “segment”, “merge”, “refine”, and “compute attributes”. The “segment” and “merge” steps of this task were applied to divide the images into segments corresponding to real-world objects and to solve over-segmentation problems the adjacent segments were merged on the basis of their brightness values. (*ii*) The “*rule-based classification*” task (i.e., classification; [20]) was used to extract only the urban land covers objects which were then exported onto raster images. For this task, the following criteria were used: (*i*) color contrast with a weight of 0.5, (*ii*) band ratio with a weight of 0.2 and (*iii*) spatial criterion with a weight of 0.3. The Nearest-Neighbors algorithm was selected for the classification task. These parameters were determined using a systematic trial and error approach validated, on test areas, by comparing the output image objects with the IKONOS ground truth [48].

#### 4.1.2. ISODATA Clustering

The Iterative Self-Organizing Data Analysis Technique (ISODATA) unsupervised classification method is (a) Iterative in that it repeatedly performs an entire classification (outputting a thematic raster layer) and recalculates statistics, and (b) Self-Organizing as it locates clusters with minimum user input [7,35,43]. The ISODATA clustering was applied to the 30m satellite data and to the MIVIS airborne data to verify if an unsupervised classification like ISODATA is able to cluster the three main urban units (i.e. vegetation, roofing and paving materials) occurring in the Venice city.

The ISODATA classifier was configured by imposing a range of output classes between 5 and 10 with at maximum 1000 iterations, setting a change threshold in the classes' aggregation process of 90%. The classification output classes were grouped into the three main urban units by using the classes' distribution of the IKONOS ground-truth image.

### 4.2. SAM classification

The Spectral Angle Mapper (SAM) supervised classification algorithm has been used for several studies, both working in multispectral and hyperspectral data spaces, providing appreciable results [7,25]. This algorithm allows performing a quick test on the spectral orthogonality of the urban material spectral classes [35]. SAM input spectra were derived from both Regions of Interest (vegetation spectra, not acquired during field campaigns), drawn directly on the images, and the ASD field measurements. The water occurring in the study area was masked by thresholding the Near-Infrared bands or whenever not accurate by digitizing it on the natural color composite of the imagery. The SAM algorithm was applied to the entire masked dataset by using seven spectral signatures of the main urban land covers identified for the Venice historical center: vegetation, new and weathered lateritic roof tiles, lead roof tiles, asphalt, trachyte, and limestone paving materials.

### 4.3. Spectral analyses

For this study, MIVIS and Hyperion hyperspectral datasets were further investigated to extract, whenever possible, fractional land cover information at the sub-pixel level. To this aim, Band and Material Detection Limit (BDL and MDL) analyses of the urban materials field spectra (collected by the ASD spectrometer) were assessed. Next, based on the MDL results, a Band Depth analyses was performed for both MIVIS and Hyperion reflectances. Moreover, a Linear Spectral Unmixing (LSU) procedure was applied to both hyperspectral data sets.

#### 4.3.1. Band and Material Detection Limit analyses

To investigate the opportunity of taking advantage of materials specific spectral features, the BDL was assessed. This parameter is defined, according to Kirkland et al. [22], as follow:

(1) $$\text{BDL}=\frac{CF}{\text{SNR}\sqrt{\frac{\text{BandFWHM}}{\text{SamplingInterval}}}}$$

Where, BDL [22,23] is the minimum band depth required for the detection of a given band width and center; SNR is the signal to noise ratio; CF (Confidence Factor) is the contrast relative to the SNR level that a feature should exhibit to be distinguished from background; Band FWHM is the full-width target band at the half maximum of the band depth and Sampling Interval represents the instrumental spectral sampling interval related to the given band.

Lower numbers for the BDL indicate that lower spectral contrast is required for detection. The CF influences the BDL such that a higher CF requires greater band contrast for acceptance (i.e., a CF = 1 represents a signal level comparable with noise).

The BDL values were calculated by assuming that no atmospheric attenuation influenced the data. To accomplish the BDL analysis, it is necessary to know the SNR of the analyzed sensor [14,41]. As this information cannot be modeled without specific knowledge of the instrument characteristics, the signal level was calculated on the mean spectral values obtained from the masks of the materials of interest. Thus, the SNR was so obtained by dividing for each masked material the signal by the corresponding standard deviation, on the basis of the method proposed by Smith and Curran [41].

Once the BDL was calculated, the minimum fractional abundance (*f_min_*) of the covering material, which has to be present in the pixel to be detected with the desired confidence, was calculated as follows:

(2) $$f_{\min}=\frac{d_{m}}{d}$$

where, *d_m_* is the spectral contrast of the material (i.e., BDL) in the image and *d* is the spectral contrast shown by the pure material measured in laboratory.

#### 4.3.2. Band-Depth Analysis

The detectable urban surface materials were assessed on the hyperspectral images using the Band-Depth (BD) analysis. The BD measures the spectral contrast of the absorption features with respect of its continuum. The application of the continuum removal process consists of: (a) fitting a straight line hull to represent the reflectance background using two continuum tie points on either sides of the absorption feature [9,24] and (b) dividing the spectrum by this fitted continuum line. The absorption band-depth (D) is calculated from the following formula [9]:

(3) D=1−Rb/Rc

where, *Rc* is the reflectance of the continuum at the band center and *Rb* is the reflectance at the band center.

#### 4.3.3. Linear Spectral Unmixing

The sub-pixel analysis procedure, applied to MIVIS and Hyperion hyperspectral data sets to extract fractional abundance images of the main surfacing urban materials, was the Linear Spectral Unmixing (LSU) procedure. The LSU is a widely used method to determine the proportion of constituent materials within a pixel based on the materials' spectral characteristics [39]. The LSU is analytically expressed as follows [37]:

(4) $$r=Mf_{N}+\varepsilon$$

where, *r* is the column vector of the measured spectrum with *L* spectral bands, *M* is the *N* × *L* endmember spectra matrix (*N* is the numbers of pure endmembers); *f* is the concentration vector whose components represent the endmember fraction for each endmember, *ε* is the residual error. In this model *M* is the known, while the unknown to be retrieved is the concentration *f_N_*.

MIVIS and Hyperion pixels' unmixing was performed using two or more spectral endmembers with fractions ranging from 0 to 100%. All the endmembers were used in the “constrain” LSU procedure [37]. The images of the coefficients for each of the endmembers obtained by the inversion procedure were normalized in order to obtain fractional abundance images for each material of interest.

## 5. Results and discussion

### 5.1. Image segmentation results

**Figure 4.**
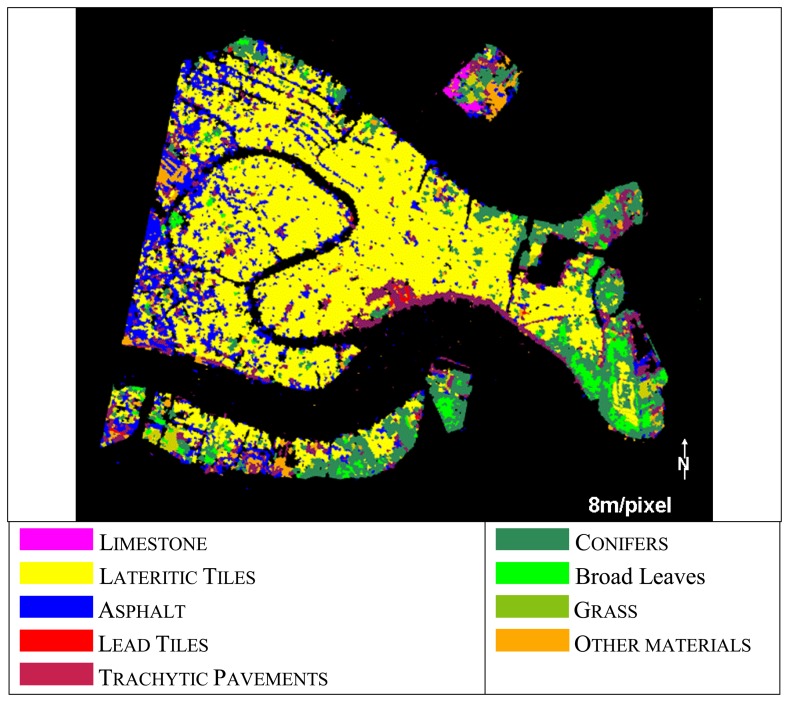
Object-oriented approach results of MIVIS data (8m/pixel).

The segmentation approach usually allows to: (a) quantify the spatial heterogeneity within the data at different scale levels; (b) delineate uniform patches; (c) implement a hierarchal structure between segments at different spatial scales. For this case study, however, the satellite spatial resolutions (30m/pixel) appear too low with respect to the urban texture and results of the object-oriented approach are extremely poor for all the satellite data. Good results, instead, were observed for the MIVIS (8m/pixel) airborne classification, for which it was even possible to discriminate different vegetation cover types, i.e. conifers, broad leaves and grass (Figure 4).

In Table 2 are shown the land cover percentage values obtained for MIVIS data by using the Object-Oriented approach as they are the only comparable to the IKONOS ground truth.

The unsupervised ISODATA output classes were grouped into three urban units (i.e. vegetation, roofing tiles and paving materials) by interpreting the classes' distribution on the imagery using as reference the IKONOS ground-truth image. Table 3 shows the percentage values attained from the ISODATA clustering for all the sensors. The vegetation class was identified with about the same percentage in each data set; in particular, MIVIS and Hyperion show the percentages closest to the values retrieved by the IKONOS interpretation. The tile roof unit is overestimated in the 30m/pixel multispectral data, while it is quite well identified in the hyperspectral datasets. The paving materials are markedly underestimated by the satellite multispectral data sets because of the spatial complexity of the study area. However both multispectral and hyperspectral imagery appear not reliable for the assessment of this unit. In fact, the buildings are to a large extent contiguous thus shadowing often the streets (only a few meters wide).

### 5.2. SAM classification results

The paving materials were trained using the spectra pertaining to the asphalt, trachyte and limestone, the roofing materials were trained using the new and old lateritic and lead tiles spectra, and the vegetation was trained with the grass, conifers and broad leaves classes.

**Figure 5.**
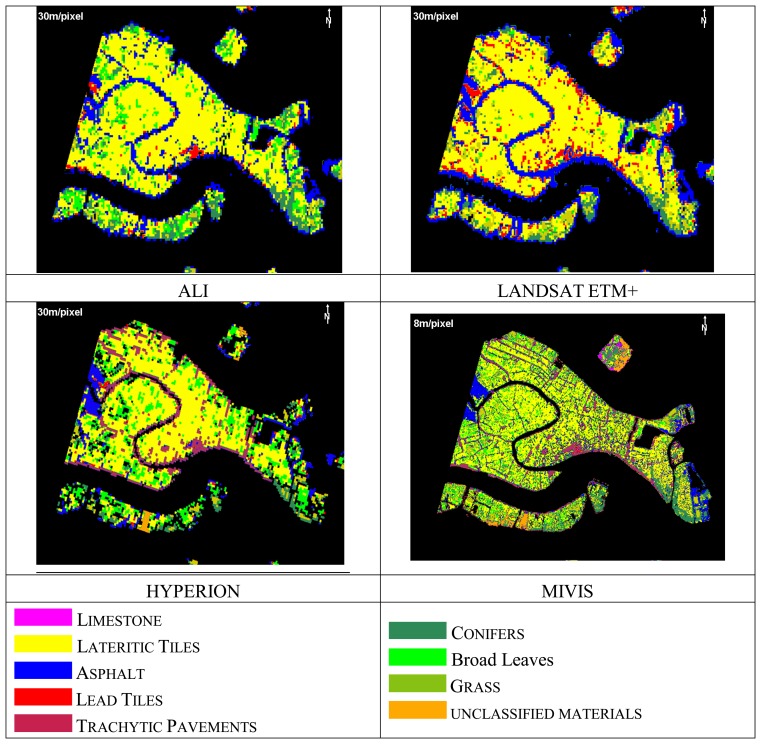
SAM classification results.

Figure 5 and Table 4 show the results of the SAM classification attained for all the sensors. SAM results show that ALI and LANDSAT ETM+ satellite data were not able to discriminate the different paving materials.

As regards the roofing materials, the retrieved amount of lateritic roofs, as combination of old and new lateritic tiles, for all the sensors is close to the IKONOS percentage; while, the lead tiles class is overestimated only by the ETM+ sensor (a percentage value of 8.0; Table 4), because too complex to be mapped at the ETM+ spectral resolution.

SAM results for ALI and LANDSAT ETM+ satellite data were not able to spectrally discriminate the paving materials. Moreover, MIVIS and Hyperion SAM classification of the asphalt and trachyte paving materials are not reliable as the spectral signature of the asphalt and the trachyte materials are both characterized by a very low reflectance (i.e. low SNR) and the lack of peculiar spectral features strongly affects their spectral separability, i.e. their detection. The only consistent results for the paving materials were achieved by MIVIS and Hyperion sensors for the limestone material, i.e. respectively a percentage value of 1.6 and 0.7 (Table 4).

### 5.3. Spectral Analyses results

The spectra of the samples collected on the field were analyzed (Figure 2) to improve the discrimination of the urban units taking advantage of their spectral features characteristics. It can be observed that: (a) the limestone paving material is characterized by absorption features in the 1.8-2.5 μm region, with a strong absorption feature centered at 2.34 mm and three weaker absorption bands at 1.85-1.97 μm, 1.97-2.00 mm and 2.12-2.16 μm [46]; (b) the trachyte paving material does not show any peculiar absorption feature; (c) the asphalt pavements are characterized by slight absorptions centered at 2.30mm and 2.35mm; (d) the lateritic tiles show an high in the Red region (0.63-0.70 mm) of the reflectance spectrum and have a peculiar peak centered at about 2.22 mm, which is typical of the silicates present in the lateritic compound; and (e) the lead oxide of the roofing tiles does not show characteristic spectral absorption features except for the peaks in the Blue (0.45-0.49 μm) and Green (0.50-0.56 mm) regions of the reflectance spectra.

In conclusion, (a) the asphalt and trachyte paving materials could be spectrally confused to each other; (b) the main absorption features of the asphalt paving correspond to the peculiar absorption feature of the limestone present as cobblestones within the asphalt paving material; (c) the weathering effects on the lateritic tiles are well discernible in the Red spectral region (old tiles show a pale red color) and in the SWIR spectral region, where a slightly deeper absorption feature at 2.22μm occurs for the new tiles.

MIVIS and Hyperion datasets were further investigated, at the sub-pixel level, in order to retrieve the real potential of the hyperspectral data in retrieving urban land cover in complex sites.

#### 5.3.1. Material Detection Limit results

Table 5 shows, for each material of interest, the *d* values (i.e. the spectral contrast shown by the pure material as measured by ASD in laboratory), the BDL and the *f_min_* values, and the minimum area required for the material to be detected by the sensor (MDA) within the pixel area (i.e. 49m^2^ for MIVIS and 900m^2^ for Hyperion).

The results shown in Table 5 highlight that only the limestone material main absorption peak centered at 2.34 mm can be detected for both Hyperion and MIVIS sensors. The 2.34 μm absorption feature is detectable if the surfacing area is higher than 14m^2^ for the MIVIS and 98m^2^ for the Hyperion sensors. However, by analyzing the study area, the only site where this result could be checked is the cemetery island (located North to the city), as the limestone is relatively abundant and it is detectable by both sensors' characteristics. Moreover, Table 5 shows that both sensors do not have the spectral or spatial characteristics to detect the spectral absorption features peculiar for identifying the new lateritic tiles and the asphalt material.

#### 5.3.2. Band-Depth and Linear Spectral Unmixing results

Following the Hyperion and MIVIS MDL results, the BD analysis was only used to detect the presence of limestone on the area of the monumental cemetery of the “San Michele” island. The main material occurring on this test area and the related percentage abundances as derived from IKONOS ground truth were: limestone (25%), cypress (8%), grass (13.5%) and lateritic roof tiles (2%).

**Figure 6.**
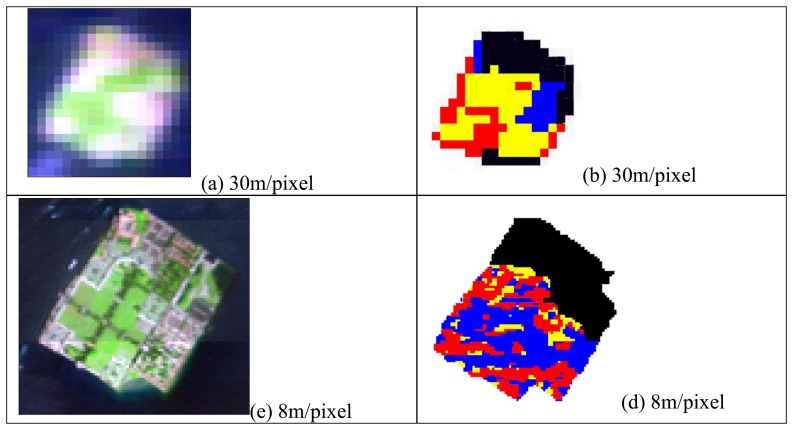
Images (a) and (c) show respectively Hyperion (zoom 12x) and MIVIS (zoom 5x) false color composite (Red=1520nm, Green=820nm, Blue=680nm) images of the cemetery island north to Venice. Images (b) and (d) show respectively Hyperion and MIVIS limestone band-depth analysis (at 2.34 μm) results.

The BD results attained for the cemetery island are shown in Figure 6. Three ranges were identified as low (red), medium (yellow) and high (blue) percentages of surfacing limestone. The red color represents the BD values ranging from 0.01 to 0.03, the yellow color from 0.03 to 0.06 and the blue color from 0.06 to 0.09. The black color refers to those pixels where the limestone peak at 2.34 did not occur. A BD value of 0.1 was calculated for the samples of pure limestone acquired by the ASD measurements.

As the BD method allowed detecting only the limestone material, in order to verify if other materials were distinguishable on the basis of the whole spectral information, a LSU procedure was applied on the same area (i.e. the cemetery island).

**Figure 7.**
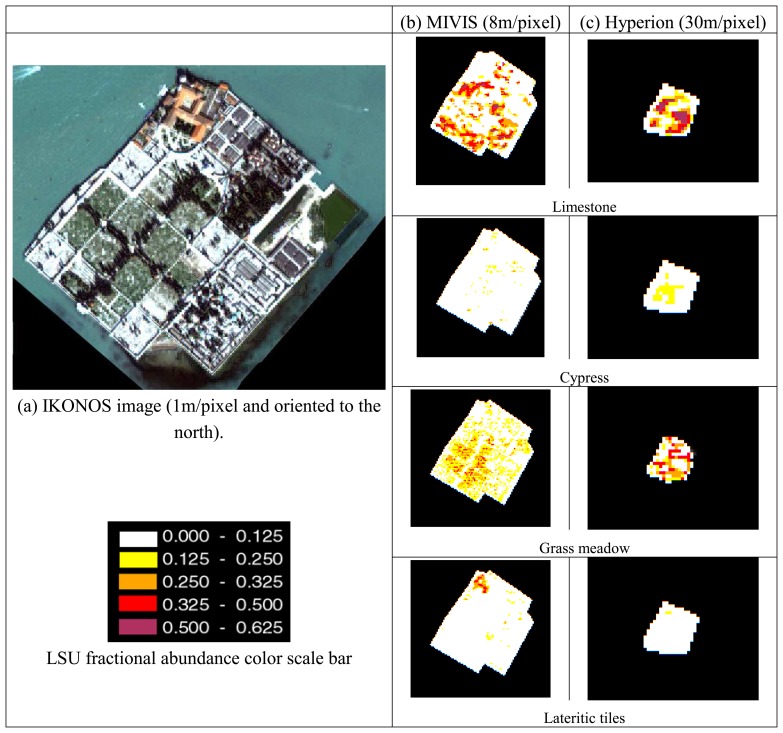
MIVIS and Hyperion fractional abundance images of the cemetery island north of Venice. IKONOS image is shown as reference. Color scale bar expresses the percentages of occurrence of the four endmembers used in the LSU analysis.

Figure 7 illustrates the results attained by applying the LSU trained with the spectra derived from the ROIs drawn on the images (i.e. cypress and grass spectra) and measured during the field campaigns (i.e. limestone and lateritic roof tiles). The fractional abundance images of the endmembers were scaled between 0-1 (a colors scale bar was adopted to depict the LSU results) and compared with those of the IKONOS ground-truth. Looking at Figure 7, the following general considerations could be made: (a) the grass class was retrieved by both sensors with a similar spatial distribution, identifying the sectors where, according to the IKONOS data, the meadow is mainly present; (b) the lateritic tiles are recognized by both sensors in the northern part of the image, where buildings are characterized by a large exposure of not weathered lateritic tiles; (c) the limestone occurrence within the island was retrieved by both MIVIS and Hyperion sensors in the area where the tombstone and the cemetery structures are made of the limestone material.

In order to evaluate the correctness of the abundance distribution of MIVIS and Hyperion LSU retrieved endmembers with respect to the IKONOS ground truth, cross correlation coefficients (*r*) were calculated and the results are reported in Table 6.

By analyzing the LSU results, it can be noticed that only MIVIS retrieved grass abundance shows the minimum acceptable level of agreement, i.e. *r*=0.75, with respect to the IKONOS distribution. Slightly lower *r* values were obtained for Hyperion vegetation endmembers due to the 30m spatial resolution that makes more complex the detection of the cypress stands.

Moreover, it has to be observed that the limestone IKONOS abundance is better correlated with the Hyperion (*r*=0.68) than MIVIS (*r*=0.45) one, thus validating the BDL analysis results, i.e. the SWIR Hyperion spectral region shows a lower *f_min_* value for the detection of the limestone material. This fact stresses that, even for the LSU procedure, the strong absorption limestone feature centered at 2.34 mm is fundamental for the material detection.

In conclusion, the results of the cross-correlation for different materials, in order to compare the unmixing accuracy using different datasets, are too low (taking into account the usual threshold of 0.75); moreover, the aforesaid comparison shows that it is difficult to proceed in this way, being the correlation index in the field of a casualty in the stochastic domain, except for the grass fractional abundance attained for the MIVIS unmixing.

## 6. Conclusions

The paper deals with the analysis of remotely sensed data recorded on the heterogeneous Venice lagoon from satellite and airborne multi/hyper-spectral sensors.

We have outlined the analysis results of a preliminary study aimed at verifying the efficiency of hyperspectral remote sensing data for mapping complex urban environments and for the production of accurate land cover maps.

Based on our experimental results, we conclude that (1) the imagery segmentation leads to an appropriate classification only for the three main urban land cover (i.e. vegetation, paving and roofing materials) for all the sensors; in particular, the object-oriented approach applied to the ALI, ETM+ and Hyperion satellite data is not able to discriminate the Venice urban land cover complexity, while better results were observed for the MIVIS (8m/pixel) airborne hyperspectral data; (2) more consistent results can be attained by using the SAM supervised spectral classification method, as it allows discriminating from a minimum of six classes (ALI and Landsat ETM+) to eight classes (Hyperion and MIVIS).

The spectral analyses, i.e. band and material detection limit, highlight that only the limestone material absorption feature at 2.34mm is exploitable for the Hyperion and MIVIS hyperspectral sensors band-depth analysis. Furthermore, the sub-pixel results attained for MIVIS and Hyperion hyperspectral datasets, highlight that only MIVIS characteristics are able to retrieve the minimum acceptable level of agreement, i.e. *r*=0.75, with respect to the IKONOS distribution, while Hyperion achieves similar results only for the limestone material (*r*=0.68).

The results of the comparison between hyperspectral and multispectral remote sensing datasets highlights that (1) Hyperion hyperspectral satellite data are capable of mapping the complex urban surface components of the Venice urban land cover with accuracy similar to the higher spatial resolution MIVIS airborne data; (2) in a complex urban context, such as that of the Venice study area, it is desirable, at the Hyperion 30m/pixel spatial resolution, to decompose pixels into their components as their sizes are smaller than the pixel size.

## Figures and Tables

**Table 1.** Characteristics of the sensors used for this study.

	**Spatial Resolution (m)**	**Bands**	**Spectral coverage (μm)**	**System of Acquisition**	**Radiometric calibration**
**(a) ALI**	10-30	10	0.4-2.4	Push-broom	L1R
**(b) HYPERION**	30	220	0.4-2.5	Push-broom	L1R
**(c) ETM+**	30	8	0.4-12.5	Push-broom	L1R
**(d) IKONOS**	1	4	0.4-0.7	Push-broom	
**(e) MIVIS**	8 (at 4000m)	102	0.4-12.7	Whisk-broom	CNR-LARA
*VIS*		20	0.4-0.83		
*VNIR*		8	1.15-1.55		
*SWIR*		64	2-2.5		
*TIR*		10	8.2-12.7		

**Table 2.** Percentages of covering materials as derived from the object-oriented segmentation procedure applied to MIVIS data.

	Conifers	Broadleaves	Grass	Lateritic STiles	Lead Tiles	Limestone	Asphalt pavements	Trachyte pavements	Other materials
MIVIS	13.9	2.3	2.3	53.9	1.6	1.4	7.7	10.4	6.6
IKONOS ground truth	17.8			52.4	1.9	1.0	5.4	12.3	9.2

**Table 3.** Retrieved ISODATA percentages of the covering materials.

	Vegetation %	Roofing Tiles %	Paving materials %	Other materials %
ALI	22.5	71.7	3	2.8
ETM+	22.3	71.12	4.22	2.36
Hyperion	18.72	60.3	15.16	5.82
MIVIS	20.31	51.23	26.27	2.19
IKONOS Ground truth	17.8	52.4	20.6	9.2

**Table 4.** Percentages of covering materials as derived from the SAM classification procedure.

	**VEGETATION**			**ROOFING MATERIALS**		**PAVING MATERIALS**			**OTHER MATERIS**
	Conifers	Broadleaves	Grass	Lateritic Tiles	Lead Tiles	Limestone	Asphalt pavements	Trachyte pavements	
**ALI**	10.3	11.3	3.2	54.2	2.2	16.7			2.1
**ETM+**	7.8	2.8	7.9	51.0	8.0	11.1			114
**Hyperion**	7.7	17.6	3.5	48.2	1.5	0.7	7.1	7.9	5.8
**MIVIS**	12.3	4.9	4.0	49.7	1.7	1.6	7.1	8.5	10.2
**IKONOS ground-truh**	17.8			52.4	1.9	1.0	5.4	12.3	9.2

**Table 5.** Values of *d*, BDL, *f_min_* and MDA calculated for the measured spectrum of the limestone and the asphalt paving materials and the new lateritic tile roofing material by using their peculiar spectral absorption features.

	**Limestone d = 0,48**		**New Lateritic Tiles d = 0,02**		**Asphalt d = 0,08**	
	MIVIS	Hyperion	MIVIS	Hyperion	MIVIS	Hyperion
**BDL %**	10,47	5,17	3,64	3,63	17,98	8,17
**f_min_%**	21,99	10,85	> 100	> 100	> 100	> 100
**MDA (m^2^)**	14	98	> pixel	> pixel	> pixel	> pixel

**Table 6.** Correlation coefficients between the fractional abundances images of MIVIS (upper table) and Hyperion (lower table) with respect to the IKONOS ground truth.

Correlation		**MIVIS**			

Coefficient		Limestone	Grass	Cypress	Tiles
	
Ground Truth	Limestone	**0.45**	0.39	0.02	0.05
	Grass	0.42	**0.75**	0.22	0.16
	Cypress	0.18	0.30	**0.43**	0.11
	Tiles	0.00	0.04	0.09	**0.42**

Correlation		**Hyperion**			

Coefficient		Limestone	Grass	Cypress	Tiles
	
Ground Truth	Limestone	**0.68**	0.32	0.16	0.22
	Grass	0.40	**0.56**	0.55	0.09
	Cypress	0.30	0.53	**0.40**	0.10
	Tiles	0.07	0.12	0.00	**0.53**

## References

[1] Bassani C., Cavalli R.M., Cavalcante F., Cuomo V., Palombo A., Pascucci S., Pignatti S. (2007). Deterioration status of asbestos-cement roofing sheets assessed by analyzing hyperspectral data. Remote Sensing of Environment.

[2] Ben-Dor E., Levin N., Saaroni H. (2001). A spectral based recognition of the urban environment using the visible and near-infrared spectral region (0.4–1.1 m). A case study over Tel-Aviv. International Journal of Remote Sensing.

[3] Berk A., Bernstein L.S., Anderson G.P., Acharya P.K., Robertson D.C., Chetwynd J.H., Adler-Golden S.M. (1998). MODTRAN Cloud and Multiple Scattering Upgrades with Application to AVIRIS. Remote Sensing of the Environment.

[4] Bianchi R., Marino C.M., Pignatti S. Airborne hyperspectral remote sensing in Italy.

[5] Bokoye A.I., Dionne P. (2004). Urban material characterization from the Hyperion hyperspectral imager: Application to downtown Montreal (Quebec, Canada).

[6] Carlson T.N., Sanchez-Azofeifa G.A. (1999). Satellite remote sensing of land use changes in and around San José, Costa Rica. Remote Sensing of Environment.

[7] Chan C.I. (2003). Hyperspectral Imaging: Techniques for Spectral Detection and Classification..

[8] Clapham W.B. (2003). Continuum-based classification of remotely sensed imagery to describe urban sprawl on a watershed scale. Remote Sensing of Environment.

[9] Clark R.N., Roush T.D. (1984). Reflectance Spectroscopy: Quantitative Analysis Techniques for Remote Sensing Applications. Journal of Geophysical Research.

[10] Collwell R.N. (1983). Manual of Remote Sensing.

[11] Datt B., McVicar T.R., van Niel T.G., Jupp D.L.B., Pearlman J.S. (2003). Preprocessing EO-1 Hyperion hyperspectral data to support the application of agricultural indexes. IEEE Transactions on Geoscience and Remote Sensing.

[12] European Space Agency Scientific Campaign Unit ESTEC (1999). Exploitation of CHRIS data from the Proba Mission for Science and Applications. Experimenters' Handbook Issue 4: Baseline Programme.

[13] Forster B.C. (1985). An examination of some problems and solutions in monitoring urban areas from satellite platforms. International Journal of Remote Sensing.

[14] Gao B. (1993). An operational method for estimating signal to noise ratios from data acquired with imaging spectrometers. Remote Sensing of Environment.

[15] Heiden U., Roessner S., Segl K., Kaufmann H. Analysis of spectral signatures of urban surfaces for their area-wide identification using hyperspectral HyMap data.

[16] Heiden U., Segl K., Roessner S., Kaufmann H. (2007). Determination of robust spectral features for identification of urban surface materials in hyperspectral remote sensing data. Remote Sensing of Environment.

[17] Hepner G.F., Chen J. Investigation of imaging spectroscopy for discriminating urban land covers and surface materials.

[18] Herold M., Gardner M., Roberts D. (2003). Spectral resolution requirements for mapping urban areas. IEEE Transactions on Geoscience and Remote Sensing.

[19] Herold M., Roberts D.A., Gardner M.E., Dennison P.E. (2004). Spectrometry for urban area remote sensing. Development and analysis of a spectral library from 350 to 2400 nm. Remote Sensing of Environment.

[20] Jensen J.R. (2005). Introductory Digital Image Processing: A Remote Sensing Perspective.

[21] Ji M., Jensen J.R. (1999). Effectiveness of subpixel analysis in detecting and quantifying urban imperviousness from Landsat Thematic Mapper Imagery. Geocarto International.

[22] Kirkland L.E., Kenneth C.H., Salisbury J.W. (2001). Thermal Infrared spectral band detection limits for unidentified surface materials. Applied Optics.

[23] Kirkland L.E., Herr K.C., Adams P.M. (2003). Infrared stealthy surfaces: Why TES and THEMIS may miss some substantial mineral deposits on Mars and implications for remote sensing of planetary surfaces. Journal of Geophysical Research.

[24] Kokaly R.F., Clark R.N. (1999). Spectroscopic determination of leaf biochemistry using band-depth analysis of absorption features and stepwise multiple linear regression. Remote Sensing of Environment.

[25] Kruse F.A., Lefkoff A.B., Boardman J.B., Heidebrecht K.B., Shapiro A.T., Barloon P.J., Goetz A. F. H. (1993). The Spectral Image Processing System (SIPS) - Interactive Visualization and Analysis of Imaging spectrometer Data. Remote Sensing of the Environment.

[26] ITT Visual Information Solutions (2008). ENVI - Environment for Visualizing Images, Version 4.4. http://www.ittvis.com/envi/.

[27] Lhermitte S., Verbesselt J., Jonckheere I., Nackaerts K., van Aardt J.A.N., Verstraeten W.W., Coppin P. (2007). Hierarchical image segmentation based on similarity of NDVI time series. Remote Sensing of Environment.

[28] Mathieu R., Aryal J., Chong A.K. (2007). Object-Based Classification of Ikonos Imagery for Mapping Large-Scale Vegetation Communities in Urban Areas. Sensors.

[29] Matthew M.W., Adler-Golden S.M., Berk A., Richtsmeier S.C., Levine R.Y., Bernstein L.S., Acharya P.K., Anderson G.P., Felde G.W., Hoke M.P., Ratkowski A., Burke H.H., Kaiser R.D., Miller D.P. (2000). Status of Atmospheric Correction Using a MODTRAN4-based Algorithm. SPIE Proceedings.

[30] Myint S.W., Lam N.S., Tyler J.M. (2004). Wavelets for urban spatial feature discrimination: comparisons with fractal, spatial autocorrelation, and spatial co-occurrence approaches. Photogrammetric Engineering and Remote Sensing.

[31] Pearlman J.S., Barry P.S., Segal C.C., Shepanski J., Beiso D., Carman S.L. (2003). Hyperion, a space-based imaging spectrometer. IEEE Transactions on Geoscience and Remote Sensing.

[32] Powell R.L., Roberts D.A., Dennison P.E., Hess L.L. (2007). Sub-pixel mapping of urban land cover using multiple endmember spectral mixture analysis: Manaus, Brazil. Remote Sensing of Environment.

[33] Pu R., Xu B., Gong P. (2003). Oakwood crown closure estimation by unmixing Landsat TM data. Int. J. Remote Sensing.

[34] Rashed T., Weeks J.R., Stow D., Fugate D. (2005). Measuring temporal compositions of urban morphology through spectral mixture analysis: Towards a soft approach to change analysis in crowded cities. International Journal of Remote Sensing.

[35] Richards J.A. (1999). Remote Sensing Digital Image Analysis..

[36] Ridd M.K. (1995). Exploring a V–I–S (vegetation–impervious surface–soil) model for urban ecosystem analysis through remote sensing: comparative anatomy for cities. International Journal of Remote Sensing.

[37] Settle J.J., Drake N.A. (1993). Linear mixing and estimation of ground cover proportions. International Journal of Remote Sensing.

[38] Small C. Scaling Properties of Urban Reflectance Spectra.

[39] Small C. (2003). High spatial resolution spectral mixture analysis of urban reflectance. Remote Sensing of Environment.

[40] Small C. (2005). A global analysis of urban reflectance. International Journal of Remote Sensing.

[41] Smith G.M., Curran P.J., Atkinson P.M., Tate N. J. (1999). Methods for estimating image signal-to-noise ratio (SNR). Advances in remote sensing and GIS analysis.

[42] Stuckens J., Coppin P.R., Bauer M.E. (2000). Integrating contextual information with per-pixel classifications for improved land cover classifications. Remote Sensing of Environment.

[43] Tou J.T., Gonzalez R.C. (1974). Pattern Recognition Principles.

[44] Toutin T. (2003). Error Tracking in Ikonos Geometric Processing Using a 3D Parametric Model. Photogrammetric Engineering and Remote Sensing.

[45] Ungar S. G., Pearlman J. S., Mendenhall J. A., Reuter D. (2003). Overview of the Earth Observing One (EO-1) mission. IEEE Transactions on Geoscience and Remote Sensing.

[46] Van Der Meer F.D., De Jong S. (2003). Imaging Spectrometry, Basic Principles and Prospective Applications.

[47] Vermote E.F., El Saleous N., Justice C.O., Kaufman Y.J., Privette J.L., Remer L. (1997). Asbestos: Geology, Mineralogy, Mining, and Uses. U.S. Department of the Interior.

[48] Wu C. (2004). Normalized spectral mixture analysis for monitoring urban composition using ETM+ imagery. Remote Sensing of Environment.

